# Direct and green repairing of degraded LiCoO_2_ for reuse in lithium-ion batteries

**DOI:** 10.1093/nsr/nwac097

**Published:** 2022-05-18

**Authors:** Junxiong Wang, Qi Zhang, Jinzhi Sheng, Zheng Liang, Jun Ma, Yuanmao Chen, Guangmin Zhou, Hui-Ming Cheng

**Affiliations:** Shenzhen Geim Graphene Center, Tsinghua-Berkeley Shenzhen Institute & Tsinghua Shenzhen International Graduate School, Tsinghua University, Shenzhen 518055, China; Frontiers Science Center for Transformative Molecules, School of Chemistry and Chemical Engineering, Shanghai Jiao Tong University, Shanghai 200240, China; Shenzhen Geim Graphene Center, Tsinghua-Berkeley Shenzhen Institute & Tsinghua Shenzhen International Graduate School, Tsinghua University, Shenzhen 518055, China; Shenzhen Geim Graphene Center, Tsinghua-Berkeley Shenzhen Institute & Tsinghua Shenzhen International Graduate School, Tsinghua University, Shenzhen 518055, China; Frontiers Science Center for Transformative Molecules, School of Chemistry and Chemical Engineering, Shanghai Jiao Tong University, Shanghai 200240, China; Shenzhen Geim Graphene Center, Tsinghua-Berkeley Shenzhen Institute & Tsinghua Shenzhen International Graduate School, Tsinghua University, Shenzhen 518055, China; Frontiers Science Center for Transformative Molecules, School of Chemistry and Chemical Engineering, Shanghai Jiao Tong University, Shanghai 200240, China; Shenzhen Geim Graphene Center, Tsinghua-Berkeley Shenzhen Institute & Tsinghua Shenzhen International Graduate School, Tsinghua University, Shenzhen 518055, China; Shenzhen Geim Graphene Center, Tsinghua-Berkeley Shenzhen Institute & Tsinghua Shenzhen International Graduate School, Tsinghua University, Shenzhen 518055, China; Faculty of Materials Science and Engineering / Institute of Technology for Carbon Neutrality, Shenzhen Institute of Advanced Technology, Chinese Academy of Sciences, Shenzhen 518055, China

**Keywords:** spent cathodes, lithium-ion batteries, LiCoO_2_, direct repair, deep eutectic solvent, lattice distortion

## Abstract

Traditional recycling processes of LiCoO_2_ rely on destructive decomposition, requiring high-temperature roasting or acid leaching to extract valuable Li and Co, which have significant environmental and economic concerns. Herein, a direct repairing method for degraded LiCoO_2_ using a LiCl–CH_4_N_2_O deep eutectic solvent (DES) was established. The DES is not used to dissolve LiCoO_2_ but directly serves as a carrier for the selective replenishment of lithium and cobalt. Replenishment of lithium restores LiCoO_2_ at different states of charge to a capacity of 130 mAh/g (at 0.1 C rate), while replenishing the cobalt increases the capacity retention rate of 90% after 100 cycles, which is comparable to pristine LiCoO_2_. The DES is collected and reused multiple times with a high repair efficiency. This process reduces energy consumption by 37.1% and greenhouse gas emissions by 34.8% compared with the current production process of LiCoO_2_, demonstrating excellent environmental and economic viability.

## Teaser

This work provides a novel direct recycling method based on a sustainable lithium-containing deep eutectic solvent to regenerate degraded cathode materials of spent lithium-ion batteries.

## INTRODUCTION

There are >100 000 tons of end of life (EOL) lithium-ion batteries (LIBs) produced from discarded portable electronic devices worldwide every year [1]. LiCoO_2_ is the dominant battery cathode material for these portable devices because of its stable performance and high specific volume capacity, accounting for ∼30% of the weight of the entire battery [2]. The Co in LiCoO_2_ is a harmful and expensive metal, which infringes the human skin and the respiratory system [3,4]. The improper disposal of EOL LIBs causes serious environmental pollution and is a huge waste of valuable metal resources [5–7].

Extensive efforts have been made to recover Li and Co from degraded LiCoO_2_ that use pyrometallurgical and hydrometallurgical processes and have recently been commercialized [8]. In general, the pyrometallurgical process requires high-temperature reduction roasting to decompose the stable LiCoO_2_ into mixed alloys for further extraction and this consumes a large amount of energy [9]. The high-temperature reduction step is substituted by acid leaching for the decomposition of LiCoO_2_ in the hydrometallurgical process, followed by precipitation, ion exchange or electrodeposition to extract Li and Co at lower temperatures [10,11]. However, a large amount of wastewater that needs further treatment is produced due to the high liquid/solid ratio during the leaching process [12]. The extensive use of acids, reduction reagents, precipitation agents and other reagents needed for this also increases the cost of the entire process [13]. The existing pyrometallurgical and hydrometallurgical processes therefore have significant problems in terms of economic feasibility and environmental friendliness. As a result, an environmentally friendly recycling process for degraded LiCoO_2_ with few steps and lower cost is urgently needed because the annual production of spent portable LIBs is predicted to increase to 180 000 tons by 2023 [1].

The direct regeneration/repair of degraded cathode materials without structural destruction to the atomic level has therefore been proposed as a promising strategy to achieve a simpler, greener and more cost-effective recycling route [14,15]. Solid-state sintering is the most widely used direct regeneration method that mixes degraded LiCoO_2_ powder with a certain amount of lithium salts, mainly Li_2_CO_3_, at ∼900°C in air [16,17]. The amount of lithium salt must be precisely controlled based on the lithium loss in the cathodes since excess lithium salt would be difficult to remove. In addition, chemical re-lithiation processes, such as directly immersing degraded LiCoO_2_ in lithium-containing solutions using a hydrothermal process, have also been reported [18,19]. The process requires a temperature of ≤180°C and high pressure, which hinders its widespread use. Another method based on an electrochemical route uses Li_2_SO_4_ as a Li^+^ resource that could replenish lithium in degraded LiCoO_2_, but the process must be operated in a specific three-electrode device [20]. However, the direct regeneration/repair methods discussed above mainly focus on replenishing lithium, while the loss of cobalt caused by dissolution during long-term cycling receives less attention. In fact, the dissolution of cobalt has a critical impact on the capacity decay and cycling stability of a LiCoO_2_ cathode [21,22].

An ideal direct regeneration/repair method should therefore meet the following requirements: (i) it must operate in solution with no need for high temperature and pressure; (ii) the solvent carrier must have selectivity for ion transmission, which is beneficial to the simultaneous replenishment of lithium and cobalt; (iii) the reagent should be low-cost; and (iv) it should have a wide application range for different kinds of cathodes. To meet these requirements, a green deep eutectic solvent (DES) has attracted our attention. This solvent has recently been used for dissolving degraded cathodes to allow the extraction of Li^+^ and Co^2+^ [23–26]. However, these investigations can be regarded as improved hydrometallurgical processes with the utilization of less acid. It will be much more meaningful if DES could be developed for direct regeneration of degraded LiCoO_2_.

Here, we report a direct repair method for completely degraded LiCoO_2_ using a sustainable lithium-containing DES. The degraded LiCoO_2_ was treated in a LiCl–CH_4_N_2_O (urea) DES under ambient pressure. The DES acts as a carrier facilitating the selective transport of lithium and cobalt to directly repair the degraded LiCoO_2_, rather than dissolving the LiCoO_2_ to give Li^+^ and Co^2+^ ions for extraction. The regeneration/repair process was optimized to guarantee an effective supply of lithium and cobalt for the degraded structure to restore it to an ideal electrochemical performance. The method shows excellent sustainability because the LiCl–CH_4_N_2_O DES can be collected and reused without the generation of hazardous waste. The direct repair method enabled by DES is relatively short with low energy consumption and few emissions, showing great potential for large-scale practical applications.

## RESULTS

### Design and formation of LiCl–CH_4_N_2_O DES

**Figure 1.**
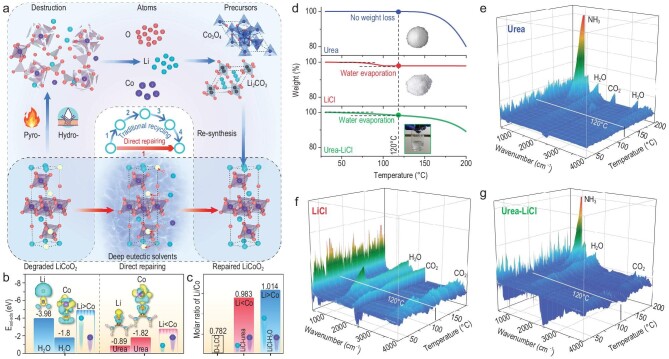
(a) Schematic of traditional recovery processes and the proposed direct repair process. (b) The adsorption energies of lithium and cobalt on solvent molecules in H_2_O and urea-based solutions. (c) The molar ratios of Li/Co in D-LCO and different LiCoO_2_ repaired in LiCl–urea and LiCl–H_2_O. (d) TG curves of urea, LiCl and urea–LiCl DES. (e–g) TG-FTIR results of (e) urea, (f) LiCl and (g) urea–LiCl DES.

Traditional recovery processes generally consist of the destruction of cathode materials into their atomic components, followed by the extraction of lithium and cobalt and a resynthesis process. The entire process is complex with relatively high cost. We have developed a nondestructive direct repair process based on LiCl–CH_4_N_2_O DES, which is much simpler than previous recovery processes (Fig. 1a). DES has a number of advantages, such as being green, nontoxic and low-cost, and having excellent solubility. However, the key point making it possible for the direct re-lithiation of degraded LiCoO_2_ (D-LCO) is its similarity to ionic liquids, which has proven to be effective for the direct re-lithiation of NCM 111 [27]. We chose urea, a cost-effective and commonly used hydrogen bond donor in eutectic solvents and calculated the adsorption energies of Li^+^ ions and Co^2+^ ions in liquid urea [28,29]. As mentioned above, the current direct re-lithiation processes merely focuses on the replenishment of lithium by a hydrothermal process at high pressure and ignores the loss of cobalt, which plays an equally critical role in the failure of LiCoO_2_ [18,21]. We found that the adsorption energy of Li^+^ ions by urea molecules is significantly smaller (−0.89 eV) than that for Co^2+^ ions (−1.82 eV), which is opposite to the results in water (adsorption energies between water molecules and Li^+^: −3.98 eV, Co^2+^: −1.80 eV, Fig. 1b). As a result, we speculated that a higher adsorption energy with solvent molecules led to priority in the order of ion diffusion in the solution. Following this assumption, the adsorption energy of Li^+^ on water molecules in an aqueous solution is much higher than that of Co^2+^, leading to the preferential diffusion of Li^+^ into LiCoO_2_. In sharp contrast, Co^2+^ ions have stronger affinity than Li^+^ with urea molecules, which indicates that Co^2+^ would be preferentially transferred from a solution to degraded LiCoO_2_. Moreover, the re-lithiation process usually occurs in a Li^+^-rich environment with a minimal Co^2+^ concentration due to the high cost of Co-containing reagents, and this also limits the supply of Co^2+^. Therefore, to repair completely degraded LiCoO_2_ under ambient pressure, a supply of Co^2+^ should be considered the first priority and urea is thus an ideal carrier. To form a DES with CH_4_N_2_O, we chose low-cost LiCl as the lithium source [30,31]. The eutectic point can be reduced to <120°C by tuning the molar ratio between LiCl and CH_4_N_2_O, which is lower than that of most current hydrothermal regeneration processes.

To verify the simulation results, the D-LCO was treated in an aqueous LiCl solution (denoted D-LCO-R-W) and a lithium-containing DES (denoted D-LCO-R-H) with the same concentrations of Li^+^ and Co^2+^. The Li/Co molar ratios in D-LCO-R-W and D-LCO-R-H were then examined. The D-LCO-R-W sample has a Li/Co ratio of 1.014, with adequate Li and insufficient Co, indicating that the replenishment of Li^+^ takes priority over Co^2+^, whereas in the D-LCO-R-H sample, the Li/Co ratio is 0.983, implying that cobalt would be adequately replenished (Fig. 1c). This result is consistent with the above simulation. Specifically, the Co^2+^ ions have greater adsorption energy than Li^+^ on urea molecules in solution, so Co^2+^ ions are preferentially transferred from the solution into the D-LCO. To investigate the electrochemical performance, both samples were heated under the same condition (850°C, 2 h) and used as cathodes for comparison in battery testing. D-LCO-R-W has an initial capacity close to that of pristine LiCoO_2_ (P-LCO) at 0.1 C (134.4 mAh/h); however, its capacity decays drastically under higher current densities during rate capability tests and a subsequent cycling test (Supplementary Fig. 1a and b). These results indicate that although lithium can be supplemented to the D-LCO under ambient pressure, the cobalt loss was not effectively replenished. The structure of the D-LCO cannot be completely restored by supplementation with lithium alone and sufficient supplementation of cobalt must be considered with high priority in the LiCoO_2_ repair process.

In addition to the above ion transport properties, the thermal stability and sustainability of the DES should also be considered. According to the thermogravimetric (TG) curves, the DES shows almost no weight loss except for a little water evaporation caused by water absorbed in the LiCl (Fig. 1d). This result is further confirmed by thermogravimetric-Fourier transform infrared (TG-FTIR) curves. Urea cannot be decomposed to produce ammonia until it is heated to 170°C, while LiCl does not produce any harmful gas even when it is heated to 200°C (Fig. 1e and f). Only H_2_O and CO_2_ were detected in the gas product when the as-formed DES was heated to 120°C (Fig. 1g). Therefore, we can infer that no redox reaction takes place during the formation of the DES and the composition of the DES remains unchanged after heating, which facilitates the recycling of the DES.

### Properties and electrochemical performance of the repaired LiCoO_2_

**Figure 2.**
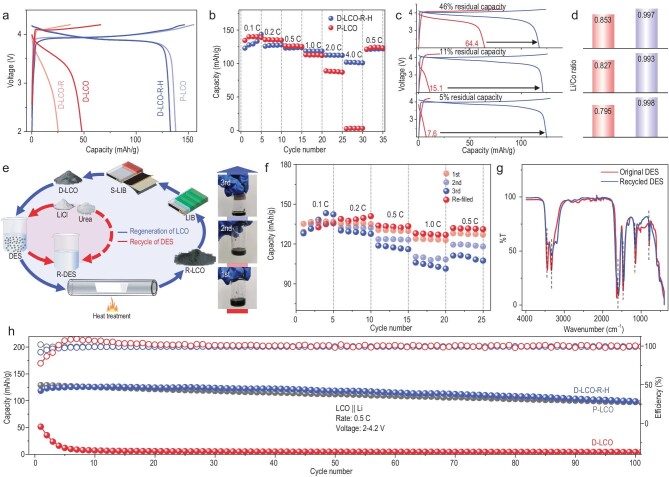
(a) The charging and discharging curves of different types of LiCoO_2_. (b) Rate capability of the different types of LiCoO_2_. (c) The charging and discharging curves of degraded LiCoO_2_ with different SOCs and the corresponding regenerated LiCoO_2_. (d) The Li/Co molar ratio of degraded LiCoO_2_ with different SOCs and the corresponding regenerated LiCoO_2_. (e) Schematic of the recycling of both the LiCoO_2_ cathode and DES. (f) Rate capabilities of repaired LiCoO_2_ after using recycled DES for one, two or three repair cycles. (g) FT–IR curves of the original and recycled DES. (h) Cycling performance of D-LCO, D-LCO-R-H and P-LCO.

D-LCO has a limited capacity of only ∼50 mAh/g in the first cycle, which rapidly decays to zero (Fig. 2a and h). In sharp comparison, the electrochemical performance of D-LCO-R-H has mostly returned to its original state with a high initial capacity and stable cycling performance. It has a capacity of 133.1 mAh/g at a 0.1 C rate, which is similar to that of P-LCO (134.4 mAh/g). The repaired LiCoO_2_ without Mg^2+^ doping shows a capacity comparable to that of P-LCO, but the cycling stability is slightly worse than that of P-LCO (Supplementary Fig. 2a). To improve the rate capability and cycling stability, the cathode material was doped with a trace amount of Mg^2+^ during annealing and the resulting D-LCO-R-H has a better rate performance at high current rates (2 C, 4 C) than P-LCO (Fig. 2b). For example, D-LCO-R-H has a capacity of 101.8 mAh/g at 4 C, while P-LCO has essentially no capacity (2.9 mAh/g) at 4 C. For the cycling performance, the capacity of D-LCO-R-H does not fade during the first 50 cycles and the capacity retention is ∼90% after 100 cycles at 0.5 C, which is identical to the P-LCO. The D-LCO-R-H demonstrates performance close to that of P-LCO at higher cut-off voltages, such as 4.3 V, and at higher temperature (60°C) (Supplementary Figs 3 and 4). There is no significant difference in coulombic efficiency during cycling (>99.5%) between the different samples, indicating that the D-LCO has been effectively repaired in terms of electrochemical performance. If the starting material was LiCoO_2_ with a higher residual capacity (∼90 mAh/g) and a less-damaged structure, the repaired LiCoO_2_ without Mg^2+^ doping could show cycling performances similar to those of P-LCO (Supplementary Fig. 2b). We also treated D-LCO in a lithium-containing DES without the addition of Co^2+^ and the resulting repaired LiCoO_2_ showed a slightly lower capacity of 124.5 mAh/g and a worse capacity retention of 67% after 50 cycles (Supplementary Fig. 5c). This result further confirms the importance of cobalt to the performance of LiCoO_2_.

To verify the versatility of this direct regeneration method, we used a chemical delithiation method to prepare and simulate D-LCOs at different states of charge (SOCs) [32]. The Li/Co ratios are 0.853, 0.827 and 0.795, and the corresponding capacities in the first cycle are 64.4, 15.1, and 7.6 mAh/g, respectively (Fig. 2c and d). The crystal structure of LiCoO_2_ is mostly destroyed and the particles gradually decrease with obvious microcracks as the degree of delithiation increases (Supplementary Figs 6 and 7). After repair, the surface of the three samples of LiCoO_2_ crystals became smooth again and the microcracks were completely healed (Supplementary Fig. 8). The capacities of all three samples of LiCoO_2_ at different SOCs are restored to ∼125 mAh/g after repair, which is comparable to that of pristine LiCoO_2_ (Supplementary Fig. 9). In summary, it has been demonstrated that this proposed method is applicable to LiCoO_2_ with various capacities and there is no need to adjust the proportion of Li^+^ salt to match the degraded LiCoO_2_ with different SOCs as has been done in previous solid-sintering methods [16]. This feature is crucial for the further exploitation of this regeneration method.

The reuse of reagents is another key factor in realizing a sustainable regeneration process. We collected the DES after each regeneration process and reused it three times (Fig. 2e). The D-LCO-R-H using recycled DES has an almost identical α-NaFeO_2_-layer structure with the space group-*R*3*m* to that of the P-LCO according to X-ray diffraction (XRD) patterns (Supplementary Fig. 10). The discharging capacities of D-LCO-R-H treated by the recycled DES are ∼130 mAh/g at 0.1 C (Supplementary Fig. 11), similar to that of the P-LCO. Their rate capabilities are also tested, giving slightly lower capacities of ∼110 mAh/g at 0.5 C after the third usage (Fig. 2f). The original DES and recycled DES are also compared using Fourier transform infrared (FT–IR) spectroscopy and are found to be almost identical (Fig. 2g). The characteristic peaks are all related to urea. There was certainly a loss in the quality of the DES after each use and the recovery rate was ∼90% according to our measurements. A certain part of DES is attached to the D-LCO and causes a mass loss. We added LiCl and CoO to the recycled DES to restore it to the original state and the rate performance of the resulting D-LCO-R-H was improved again, delivering 135.2 mAh/g at 0.1 C and 123.9 mAh/g at 0.5 C, which are comparable to the values of P-LCO (Fig. 2f). The DES accounts for the major cost of the proposed regeneration process and the potential economic benefit of this proposed regeneration process is greatly improved because of the reuse of the DES.

### Regeneration mechanism of D-LCO using DES

**Figure 3.**
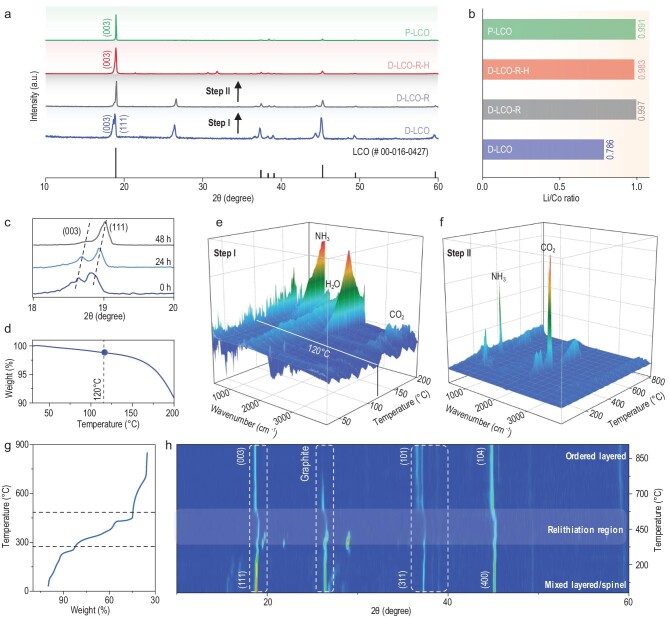
(a) XRD patterns, (b) Li/Co molar ratios of different types of LiCoO_2_. (c) XRD characteristic peaks of D-LCO treated in DES for different times. (d) TG curves of the mixture of D-LCO and DES. (e and f) TG-FTIR results of a mixture of (e) D-LCO and DES, and (f) D-LCO-R. (g) TG curve of D-LCO-R. (h) *In situ* XRD patterns of D-LCO-R at different temperatures.

A variety of characterization methods were used to explore the repair mechanism of D-LCO. The XRD pattern of D-LCO shows some miscellaneous peaks corresponding to acetylene black at ∼26° and spinel LiCoO_2_ at ∼19° (related to the (1 1 1) plane), which are not present for P-LCO (Fig. 3a). The spinel LiCoO_2_ originates from Li/Co disorder as a result of long-term cycling [33]. After two steps of regeneration, the D-LCO-R-H shows a similar layer α-NaFeO_2_-structure with the space group-*R*3*m*, as does P-LCO. Moreover, the D-LCO has a Li/Co molar ratio value of only 0.786, indicating remarkable lithium loss, which is increased to 0.997 after Li/Co refurbishment and slightly decreases to 0.983 after annealing, which is due to Li loss during high-temperature annealing. The Li/Co ratio in D-LCO-R-H is quite close to that of P-LCO (0.991), indicating that the Li and Co loss in D-LCO has been effectively restored (Fig. 3b).

The phase transition of D-LCO during the regeneration steps was carefully studied. The first step (DES treatment) in the repair process lasted for ∼48 h and the characteristic peak (0 0 3) related to layered LiCoO_2_ gradually disappeared (Fig. 3c and Supplementary Fig. 12). This means that an unexpected phase transition from layered to spinel LiCoO_2_ occurs during long-term heating at 120°C. This spinel phase is usually observed in LiCoO_2_ sintered at low temperature (LT), i.e. LT-LCO (<400°C). As mentioned above, Co ions are preferentially transported in the DES and they may therefore occupy the vacant Li sites caused by Li loss. This Li/Co disorder leads to the generation of a spinel phase. No weight loss was detected during this DES treatment step except for slight water loss (<2%) (Fig. 3d), indicating that no harmful gas was produced. The TG-FTIR result further confirms this result, as there is no other gas product except for H_2_O and CO_2_ (Fig. 3e). A certain amount of DES is attached to the surface of D-LCO, which decomposes during the second annealing step and generates ammonia (Fig. 3f). The produced ammonia is absorbed by water. The mass loss of DES is mainly attributed to this step. A weight loss is observed in D-LCO treated in DES (D-LCO-R) during the annealing step (Fig. 3g). The TG curves of D-LCO-R show that ∼67% of the total weight is lost due to the decomposition of the attached DES on D-LCO and the residual 33% of the total weight is the mass of the obtained D-LCO-R-H. Based on this ratio, 4.45 g of DES was used to treat 0.2 g of D-LCO and the attached DES on D-LCO should be ∼0.4 g, indicating that the recycling rate of DES should be ∼90%, which is very close to our previous measurement.

D-LCO-R shows a dominant phase of spinel after DES treatment, which is then converted back to an ordered layer during the annealing step (Fig. 3h). Only one main peak (1 1 1) related to spinel LiCoO_2_ at ∼19° is observed in D-LCO-R before heating to 350°C. The location of this peak remains constant in this temperature range, implying that no re-lithiation occurs. Then, another characteristic peak (0 0 3) related to layered LiCoO_2_ appears between 350 and 450°C. The emergence of a layered structure indicated that Li and Co atoms in the lattice began to rearrange. The (0 0 3) peak continues to increase, while the (1 1 1) peak decreases until only the (0 0 3) peak remains at 450°C, implying that a phase transition driven by thermal treatment takes place (Supplementary Fig. 13). The (0 0 3) peak gradually shifts to a lower angle between 450 and 600°C, indicating lattice expansion caused by Li re-intercalation. Then, the location of the (0 0 3) peak does not change as the temperature further rises to 850°C. D-LCO with a mixed phase undergoes two sequential phase transition processes including layered to spinel and spinel to layered in this repair process. This result has rarely been noticed in previous studies because most current studies are based on degraded LiCoO_2_ with a relatively higher residual capacity and complete structure [16–18].

**Figure 4.**
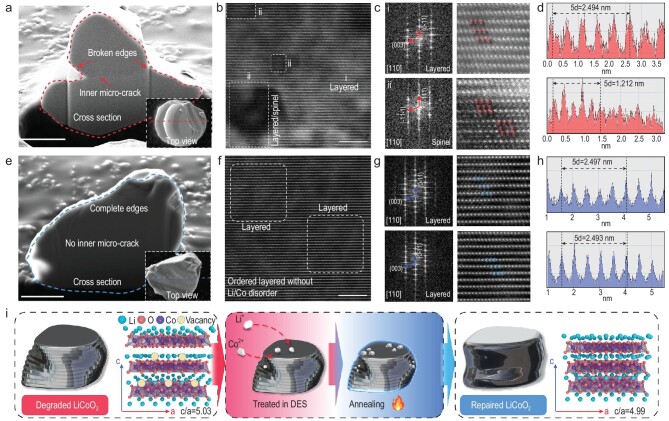
The repair mechanism of D-LCO. (a) FIB-SEM image (scale bar = 2 μm), (b) HRTEM images (scale bar = 5 nm), (c) SAED patterns, atomic arrangements and (d) the corresponding interplanar spacing of D-LCO. (e) FIB-SEM image (scale bar = 2 μm) and (f) HRTEM images (scale bar = 5 nm), (g) SAED patterns, atomic arrangements and (h) the corresponding interplanar spacing of D-LCO-R-H. (i) Schematic of the repair mechanism of D-LCO.

Fractures and microcracks can be clearly observed on the surface and cross section of the D-LCO (Supplementary Fig. 14a and Fig. 4a). A more detailed examination of microcracks was conducted using focused ion beam scanning electron microscopy (FIB-SEM) and transmission electron microscopy (TEM) (Supplementary Figs 15 and 16) and a quite different smooth surface without microcracks was seen for D-LCO-R-H (Supplementary Fig. 14b and Fig. 4e). The change in morphology is quite similar to that of chemically delithiated LiCoO_2_ (Supplementary Fig. 8). As mentioned before, the spinel LiCoO_2_ phase is observed in D-LCO (Fig. 3a), which is confirmed by Raman and selected area electron diffraction (SAED) results. A characteristic peak related to spinel LiCoO_2_ at 590 1/cm (Supplementary Fig. 17) is observed in D-LCO and a few areas containing Li/Co antisites that cause a phase transition from layered to spinel LiCoO_2_ are seen in the high-resolution transmission electron microscopy (HRTEM) image (Fig. 4b), while in D-LCO-R-H, only an ordered layered structure is seen (Fig. 4f). Detailed SAED patterns derived from fast Fourier transition and the corresponding atomic arrangement of Co atoms that shows the ordered layer and spinel structure of LiCoO_2_ are compared in Fig. 4c and g. It is clear that there are irregular atoms distributed between the Co layers in the D-LCO, indicating that the cobalt atoms have occupied the lithium site. In contrast, an ordered layered structure without any lattice distortion is observed in D-LCO-R-H. The Li/Co disorder leads to the phase transformation and the interplanar spacings also change significantly. For D-LCO, the interplanar spacing is 4.9 Å in the layered area, which decreases to 2.4 Å in the spinel area (Fig. 4d). However, a uniform interplanar spacing of 4.9 Å was measured for D-LCO-R-H (Fig. 4h). More detailed SAED patterns acquired using TEM are shown in Supplementary Fig. 18. This ordered layer lattice contributes to stabilizing the bulk diffusion of Li^+^ during battery cycling [34]. We also detected another crystal plane (3 1 1) corresponding to spinel LiCoO_2_ and measured its interplanar spacing (Supplementary Fig. 19) using HRTEM, finding it to be slightly smaller than that of the (1 0 1) plane of layered LiCoO_2_. These results show that Li/Co supplementation and reordering play a key role during microstructure repair. Part of the high-valence Co (Co^4+^) in the D-LCO is also reduced to Co^3+^ after repair, implying that a redox reaction occurred as Li^+^ re-intercalated (Supplementary Fig. 20).

By combining all the above results, we suggest the repair mechanism of D-LCO shown in Fig. 4i. The D-LCO is first treated in LiCl–CH_4_N_2_O DES with the addition of Co^2+^. Li^+^ and Co^2+^ ions are selectively transferred to the surface of D-LCO and gradually diffuse into the bulk of D-LCO to refill the vacancies in the lattice. The subsequent annealing accelerates the rearrangement of Li and Co atoms, causing the phase of spinel LiCoO_2_ to convert back to layered LiCoO_2_.

### Environmental and economic analysis

**Figure 5.**
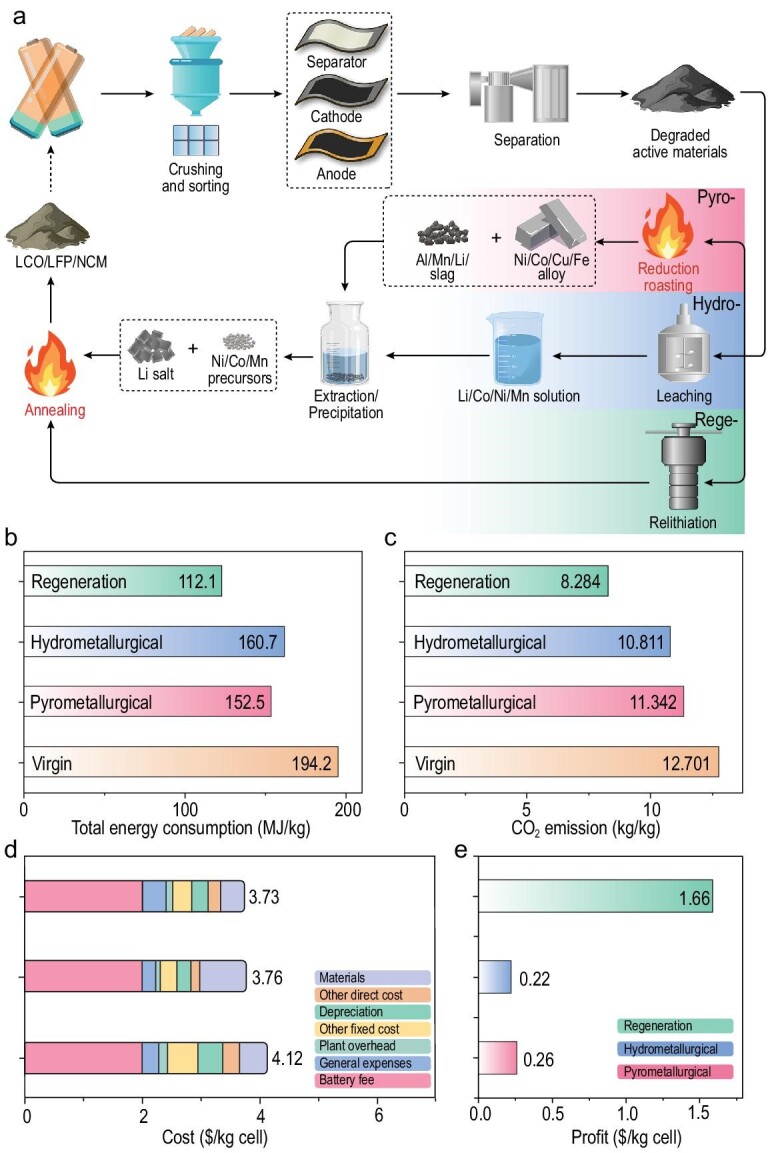
Economic and environmental analysis of this proposed direct regeneration method. (a) Brief comparison of the different recycling processes. (b) The total energy consumption, (c) the GHG emissions, (d) the cost in detail and (e) the potential benefit of the different recycling processes.

Our proposed direct regeneration method was compared with current pyrometallurgical and hydrometallurgical recycling processes for LiCoO_2_ in terms of energy consumption, greenhouse gas (GHG) emission, cost and potential benefit. The direct regeneration method has significantly fewer steps than the other two routes (Fig. 5a) [5]. Both high-temperature smelting and aqueous leaching are avoided, leading to a significant decrease in energy consumption and GHG emissions. The problem of treating acid-containing wastewater is also eliminated because the lithium-containing DES can be completely recycled after use. Specifically, the energy consumption of this direct regeneration process for D-LCO is 122.1 MJ/kg, which is much lower than that of the pyrometallurgical process (152.5 MJ/kg) and hydrometallurgical process (160.76 MJ/kg) and is only 62.9% of the energy used for the production of a virgin cathode from raw lithium ores (Fig. 5b). The same trend is found for GHG emissions, where the lowest emission (8284 kg/kg) is for the direct regeneration process, which is only 65.2% of that of virgin cathode production (Fig. 5c). Therefore, this direct regeneration method has significant advantages in energy savings and emission reduction for the production of LiCoO_2_.

The costs of the three recycling methods are also carefully compared (Fig. 5d). The cost of pyrometallurgical recycling is the highest, reaching 4.2 $/kg cell. The cost of the hydrometallurgical process is 3.8 $/kg cell and that of the direct regeneration process is 3.7 $/kg cell, which is the lowest of all due to the shorter process and lower energy consumption. For the direct regeneration method, the majority of the cost originates from the battery fees (2.0 $/kg cell), followed by the cost of materials (0.4 $/kg cell), i.e. reagent cost. The cost was considered intentionally before we investigated this method and was the reason we chose low-cost CH_4_N_2_O and LiCl for the DES. The cost of reagents is further reduced by their reuse. Due to the shorter process, lower energy consumption, lower reagent cost and high value of LiCoO_2_, this direct regeneration method produces substantial savings over using new materials for a cell. These can be as much as 1.7 $/kg cell, which is much higher than those of pyrometallurgical (0.25 $/kg cell) and hydrometallurgical recycling (0.23 $/kg cell, Fig. 5e). These results are based on the simulation results of the EverBatt 2020 model, which aims at providing guidance and is not a precise calculation. While the above estimates may not be completely accurate, the relative numbers are believed to be reasonable and have been verified by many previous studies [14,35]. The real benefit of the direct regeneration method will only be seen after a practical commercial attempt. At present, the recycling rate of spent LIBs worldwide may be <5% and the poor economic feasibility of existing recycling methods is a major obstacle to their recycling [7]. This direct regeneration method provides a promising, environmentally friendly method that is simple and has great potential benefits. More consideration should be given to the design of low-cost reusable reagents for the repair of the cathode to further improve the economic feasibility in the future, eventually reaching practical criteria on a large scale.

## DISCUSSION

We have developed a new direct regeneration method for degraded LCO using a sustainable CH_4_N_2_O–LiCl DES. The capacity of the degraded LCO could be restored to ∼130 mAh/g and cycled stably at 0.5 C. The DES could be easily collected and reused several times, making this method a more environmentally friendly strategy than conventional recycling processes, with no production of wastewater and low energy consumption. The EverBatt 2020 analysis suggests a potential benefit of 1.7 $/kg cell, indicating the high economic feasibility of the process. Further studies based on other sustainable reagents should be conducted to achieve profitable recycling of other cathode materials. The recycling of LIBs involves not only the problem of cathode treatment but also the collection and transportation of the spent LIBs and the efficient crushing and sorting of the different components, all of which are necessary to achieve practical and profitable battery recycling.

## MATERIALS AND METHODS

### Raw materials

The spent batteries were first soaked in a 0.1 M NaCl solution for 24 h to completely discharge them, followed by manual dismantling and separation into cathodes, anodes and separators. The collected cathodes were cut into small pieces and placed in a 1 M NaOH solution to dissolve the Al foil [36]. Afterward, the slurry was filtered and dried prior to heating at 500°C in air to remove the polyvinylidene fluoride (PVDF) binder [37]. The residue containing degraded LiCoO_2_ (referred to as D-LCO) and acetylene black was collected and dried. The D-LCO in this manuscript has almost no initial capacity, implying a completely destroyed structure. Another kind of D-LCO with a residual capacity of ∼90 mAh/g was compared to illustrate why Mg^2+^ doping is essential. Lithium chloride (LiCl, AR, 99.0%), urea (CH_4_N_2_O, AR, 99%), cobalt oxide (CoO, AR, 99%), magnesium fluoride (MgF_2_, AR, 99%) and *N*-methyl-pyrrolidone (NMP, AR, 99%) were purchased from Macklin Co. Ltd. Pristine LiCoO_2_ was purchased from Kluthe Co. Ltd. as a comparison material and is referred to as P-LCO.

### The repairing process

LiCl and CH_4_N_2_O were mixed in a molar ratio of 3:1 to form a DES after heating at 100°C for 1 h. The D-LCO and a small amount of CoO were added to the DES with stirring for direct repair at 120°C. Specifically, we used 0.85 g of LiCl (20 mmol) and 3.6 g of urea (60 mmol) to prepare the DES and the mass of degraded LiCoO_2_ was 0.2 g (∼2 mmol), leading to a DES:LiCoO_2_ mass ratio of 22.25:1. The content of CoO in the DES has a profound influence on the electrochemical performance of the repaired LiCoO_2_ as shown in Supplementary Fig. 5 and was eventually optimized to 5 wt%. The formed slurry was filtered and rinsed with deionized water and ethanol several times to obtain the repaired LiCoO_2_ (denoted D-LCO-R), which was subsequently dried and annealed for 2 h at 850°C in air. The residual carbon additive was also removed simultaneously. In addition, the DES treatment and annealing were separately tested to examine and compare their reparative effects. The results show that the ideal repair was only achieved by their combination (Supplementary Fig. 21). In addition, a small amount of Mg^2+^ was doped in the repaired LiCoO_2_ during annealing to promote its cycling stability [38,39]. The repaired LiCoO_2_ was denoted D-LCO-R-H (repair and heated). The filtrate, containing water, ethanol, LiCl and CH_4_N_2_O, was also collected and heated at 100°C to evaporate the water and ethanol, and obtain recycled DES for a second use. The collected DES was used three times and all the corresponding repaired LiCoO_2_ was collected and characterized. In addition, a LiCl aqueous solution was used to repair D-LCO instead of the above DES and the obtained LiCoO_2_ was denoted D-LCO-R-W.

## Supplementary Material

nwac097_Supplemental_File
