# Supplementary material for: Direct and green repairing of degraded LiCoO2 for reuse in lithium-ion batteries
Source: Natl Sci Rev. 2022 May 18;9(8):nwac097. doi: 10.1093/nsr/nwac097 (PMC9385464; doi:10.1093/nsr/nwac097)
Supplement: nwac097_Supplemental_File [file nwac097_supplemental_file.docx]

**Supplementary information**

**Direct and green repairing of degraded LiCoO_2_ for reuse in lithium- ion batteries**

Junxiong Wang ^#, a, b^, Qi Zhang ^#, a^, Jinzhi Sheng ^#, a^, Zheng Liang *^, b^, Jun Ma ^a^, Yuanmao Chen ^b^, Guangmin Zhou *^, a^, Hui-Ming Cheng*^, a, c^

^a^ Shenzhen Geim Graphene Center, Tsinghua-Berkeley Shenzhen Institute & Tsinghua Shenzhen International Graduate School, Tsinghua University, Shenzhen 518055, China

^b^ Frontiers Science Center for Transformative Molecules, School of Chemistry and Chemical Engineering, Shanghai Jiao Tong University, Shanghai 200240, People’s Republic of China

^c^ Faculty of Materials Science and Engineering / Institute of Technology for Carbon Neutrality, Shenzhen Institute of Advanced Technology, Chinese Academy of Science, Shenzhen 518055, China

* Corresponding authors: Zheng Liang (Email:[liangzheng06@sjtu.edu](mailto:liangzheng06@sjtu.edu).cn); Guangmin Zhou (Email: [guangminzhou@sz.tsinghua.edu.cn](mailto:guangminzhou@sz.tsinghua.edu.cn)), Hui-Ming Cheng (Email: [cheng@imr.ac.cn](mailto:cheng@imr.ac.cn))

(Total 1 text, 21 figures)

**Supplementary experimental information**

*Electrochemical performance measurements*

Different types of LiCoO_2_, together with acetylene black (AB, Denka Black Li-400) were carefully mixed and ground in a mortar for 30 minutes, and the mixture was dropped into NMP with the addition of a PVDF binder in LiCoO_2_: AB: PVDF mass ratios of 8:1:1. The obtained slurry was coated onto an Al foil, followed by vacuum drying at 80 °C for 12 h to produce the cathode plate that was cut into round disks (12 mm diameter) for assembly into coin cells (Type:CR2032). Metallic lithium chips were selected as the anode and 1 M LiPF_6_ in ethylene carbonate (EC)/dimethyl carbonate (DMC) in a volume ratio of 50/50 was used as the electrolyte. The mass loading of active materials was controlled at approximately 5 mg/cm^2^. Electrochemical performance, including rate capability and cycling stability was studied using a Lanhe battery test system. The current rate for the rate capability tests ranged from 0.1 C to 4 C, and the cut-off voltage during charging was set at 4.2 V (versus Li/Li^+^) for all tested cells (1C=140 mAh/g). The cycling stability at the 0.5 C rate was tested immediately after the rate capability test.

*Materials characterization*

The crystalline phases of the LiCoO_2_ samples were examined by X-ray diffraction (XRD, D8 Advance) with Cu Kα radiation at a scan rate of 5° 2θ /minute. The morphology of the LiCoO_2_ samples were characterized by focused ion beam scanning electron microscopy (FIB-SEM, Hitachi SU8010), and transmission electron microscopy (TEM, FEI Tecnai G2 F30). LiCoO_2_ samples were completely dissolved in a mixture of 5 M HNO_3_ and 30 v/v% H_2_O_2_ to examine the molar ratio of Li/Co by inductively coupled plasma optical emission spectrometry (ICP-OES 8300, Arcos Ⅱ MV). Raman spectra of the LiCoO_2_ samples were obtained using a Horiba LabRAM HR800. The original and recycled DES were both directly characterized by Fourier transform infrared spectroscopy (FT-IR, Nicolet 50) in the liquid state. To compare the adsorption energies of Li^+^ and Co^2+^ in different solvents, including water and urea, an implicit solvation calculation based on spin-polarized DFT was conducted using a software package that incorporates solvation into the Vienna Ab-initio Simulation Package (VASP) within a self-consistent continuum model.

*Economic and environmental analysis*

The economic and environmental analyses of the different recycling processes were conducted using the EverBatt 2020 model, developed by the Argonne National Laboratory, USA. This software is designed to simulate the energy consumption, cost, greenhouse gas (GHG) emission, possible output, economic benefits and other indicators for different battery recycling methods. Pyrometallurgical recycling is usually criticized due to its high energy consumption and GHG emissions, while the high cost of reagents used in hydrometallurgical recycling is in debate. We therefore made primary comparisons including the energy consumption, GHG emission, cost and the possible benefits of the pyrometallurgical, hydrometallurgical, and the direct regeneration processes proposed here. The cost of DES was considered in economic analysis because the preparation of DES accounts for the major part of materials cost. Since DES can be recycled and reused, the recovery rate is about 80% each batch, so the material cost will be reduced by the same proportion, which is reflected in the overall cost. The production of cathodes derived from raw material was also included in the comparison, and is referred to as the virgin process. The simulation is based on the following assumptions. The battery recycling plant is located in China with an annual processing capacity of about 10,000 tons of spent LiCoO_2_ battery cells, producing 3954 tons of regenerated LiCoO_2_ corresponding to 9,800 tons of new batteries. The transportation and dismantling costs of spent batteries are basically the same for the different recycling processes, so these are not included in the economic analysis.


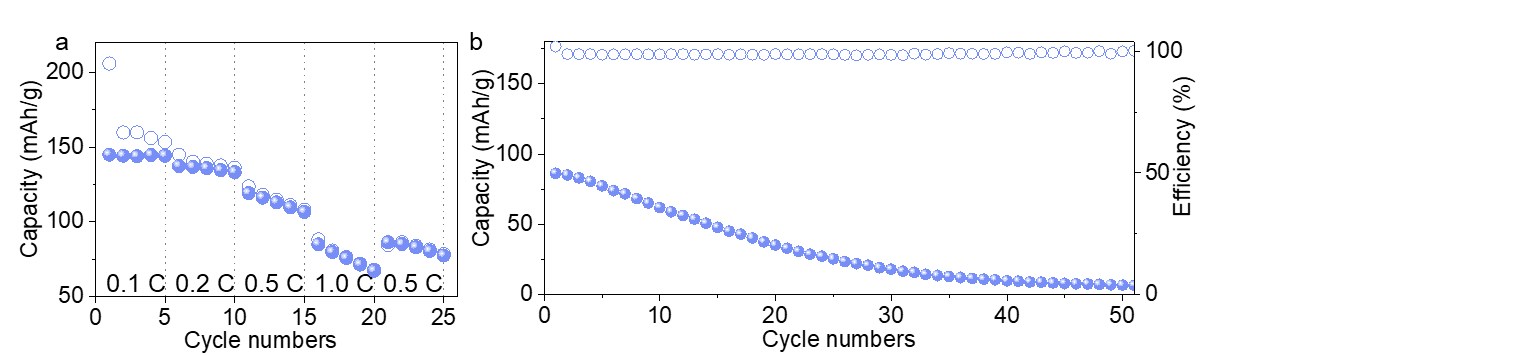


**Fig. 1 a,** Rate capabilities, and **b**, Cycling performance of LiCoO_2_ repaired in LiCl-H_2_O. (The rate capability of D-LCO-R-W is unsatisfactory, and the cycling test began right after the rate capability test. Therefore, the discharging capacity is only 80 mAh/g at the beginning of cycling test)


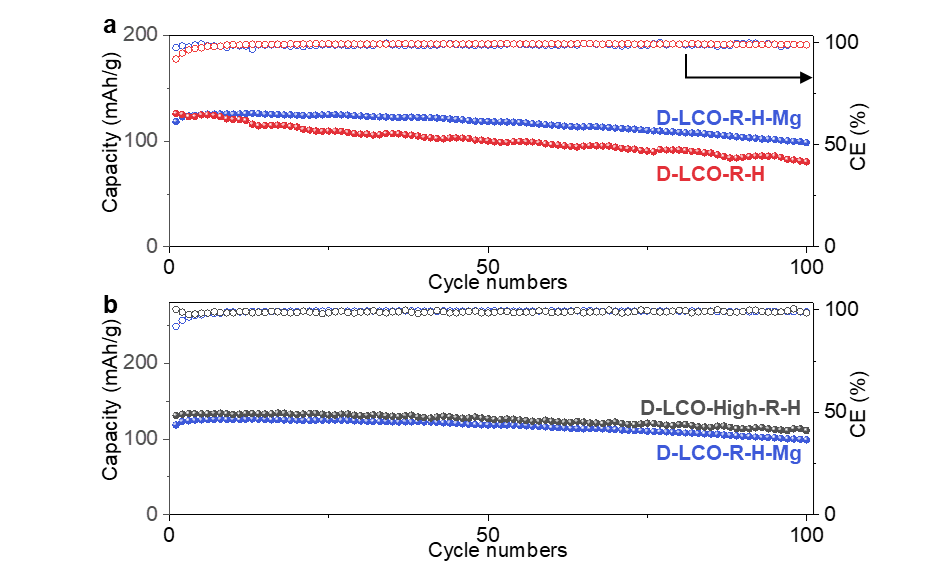


**Fig. 2** **a**, Cycling performance of repaired LiCoO_2_, D-LCO-R-H means completely degraded LiCoO_2_ using DES and annealing without Mg doping, D-LCO-R-H-Mg is the data shown in the manuscript; **b**, Cycling performance of repaired LiCoO_2_, D-LCO-R-H-Mg is the data shown in the manuscript, and D-LCO-High-R-H means repaired LiCoO_2_ derived from degraded LiCoO_2_ with higher residual capacity.


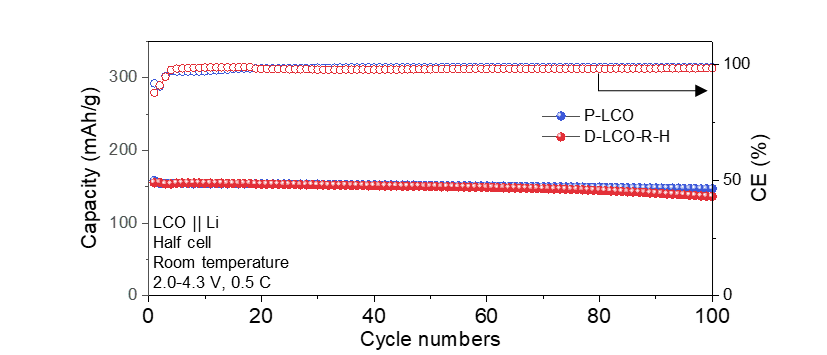


**Fig. 3** Cycling performances of D-LCO-R-H and P-LCO at a voltage range of 2.0-4.3 V.


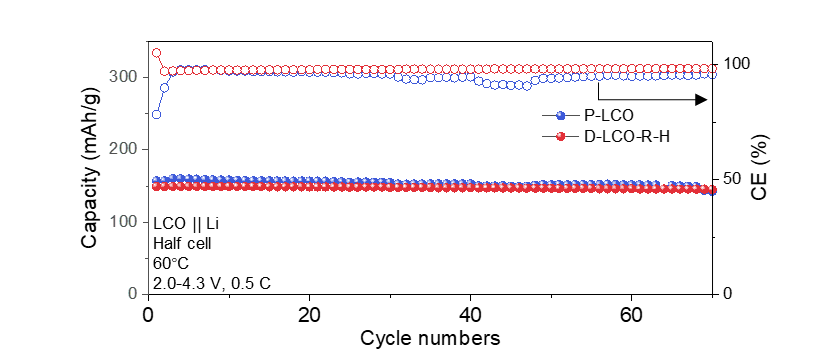


**Fig. 4** Cycling performances of D-LCO-R-H and P-LCO at a voltage range of 2.0-4.3 V under 60°C.


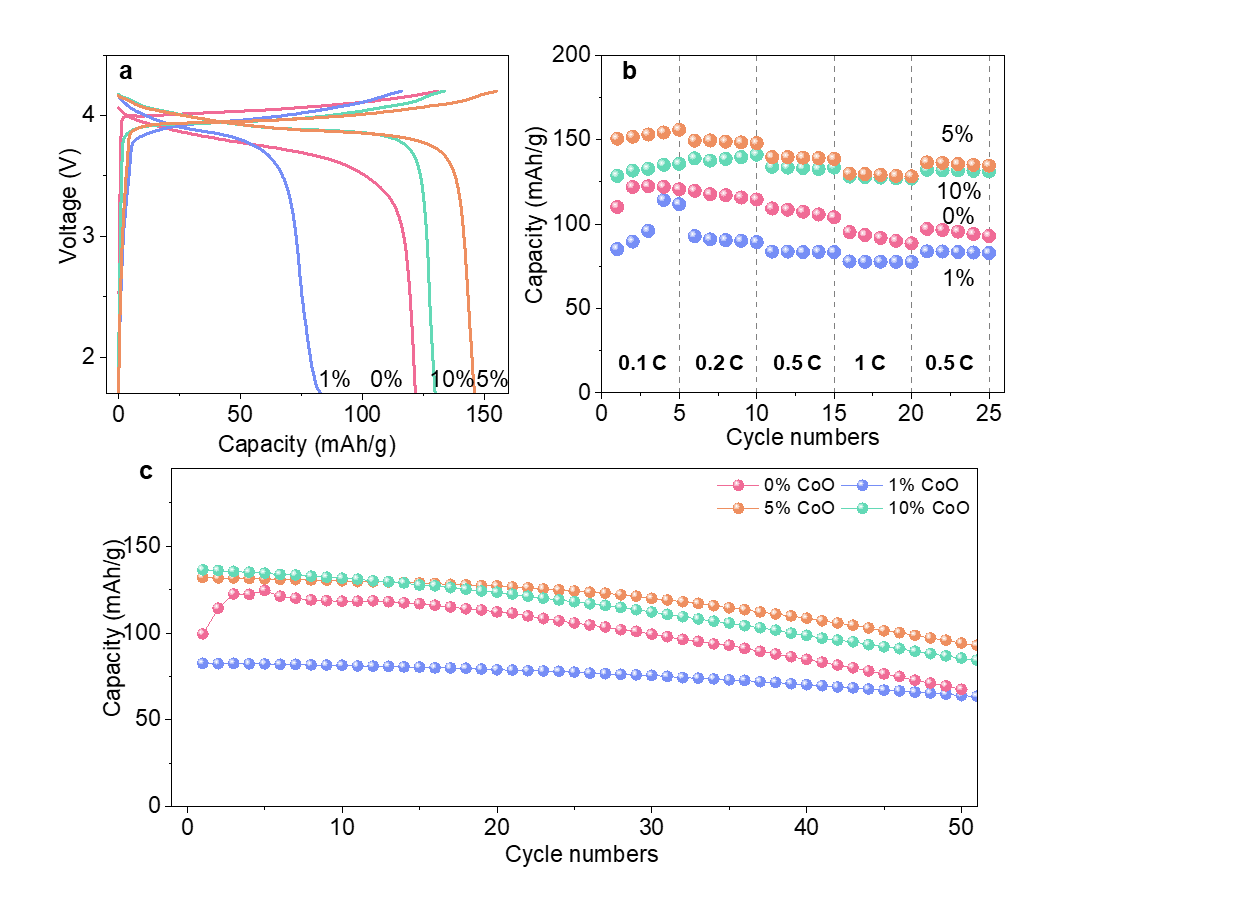


**Fig. 5 The effect of CoO content on the performance of repaired LiCoO_2_. a**, Charging and discharging curves of repaired LiCoO_2_ in DES with different CoO contents. **b**, Rate capabilities of repaired LiCoO_2_ in DES with different CoO contents. **c**, Cycling performance of repaired LiCoO_2_ in DES with different CoO contents.


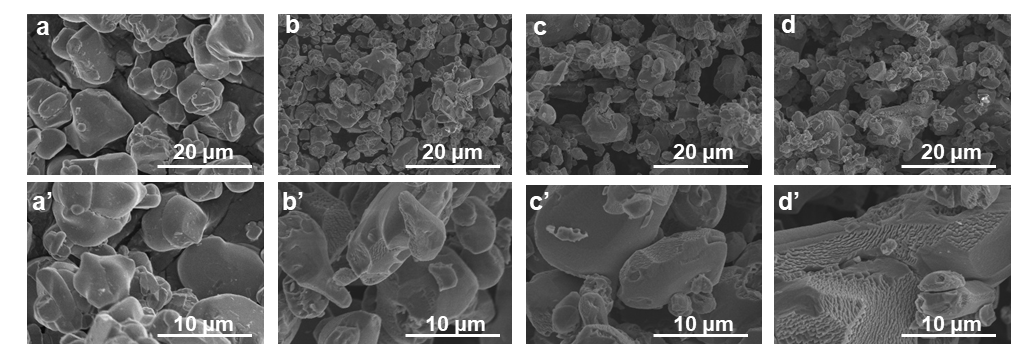


**Fig. 6 SEM images of degraded LiCoO_2_ after different de-lithiation times. a**, **a’**, pristine LiCoO_2_. **b**, **b’,** 10 mins. **c**, **c’,** 30 mins. **d**, **d’,** 60 mins.


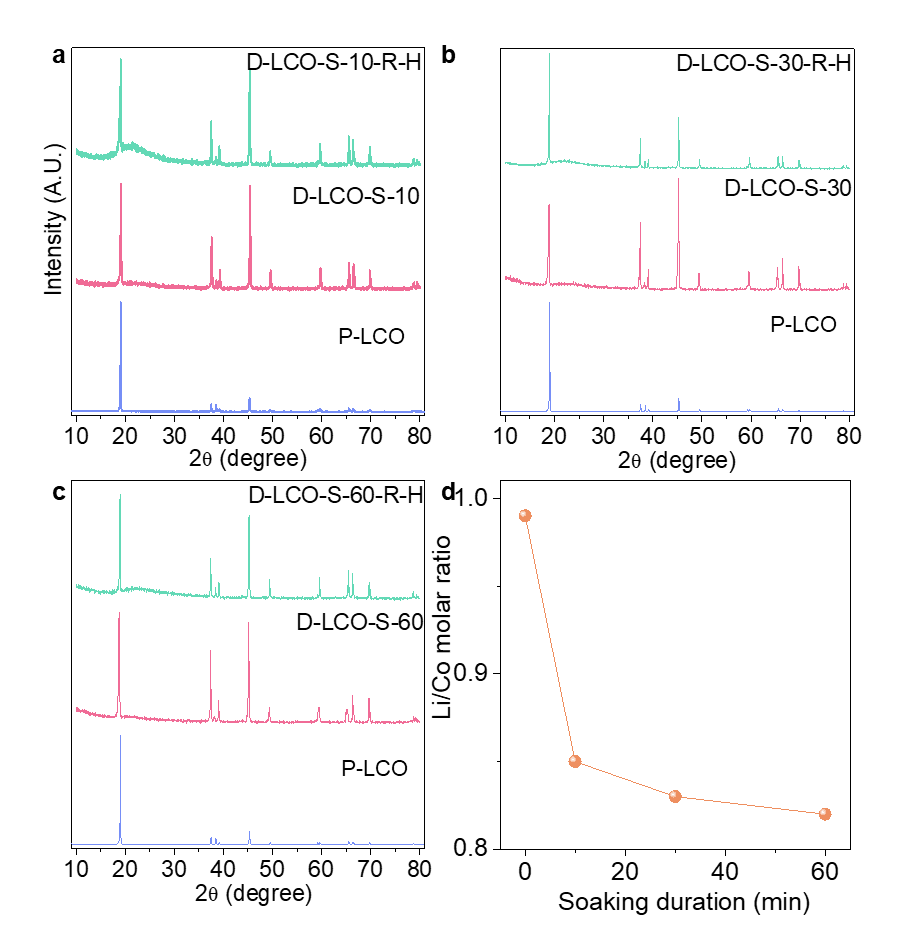


**Fig. 7 XRD patterns of different degraded LiCoO_2_ materials before and after repair. a**, 10 mins. **b**, 30 mins. **c**, 60 mins. **d**, The corresponding Li/Co molar ratio of degraded LiCoO_2_.


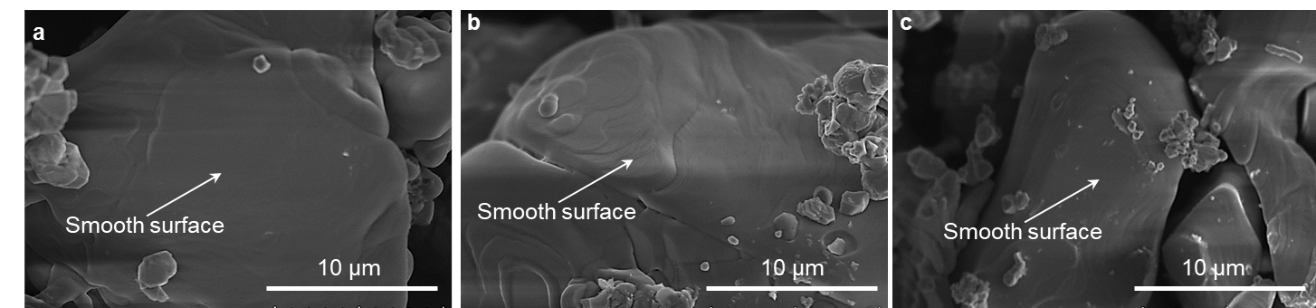


**Fig. 8** **SEM images of different degraded LiCoO_2_ materials after repair. a**, 10 mins. **b**, 30 mins. **c**, 60 mins.


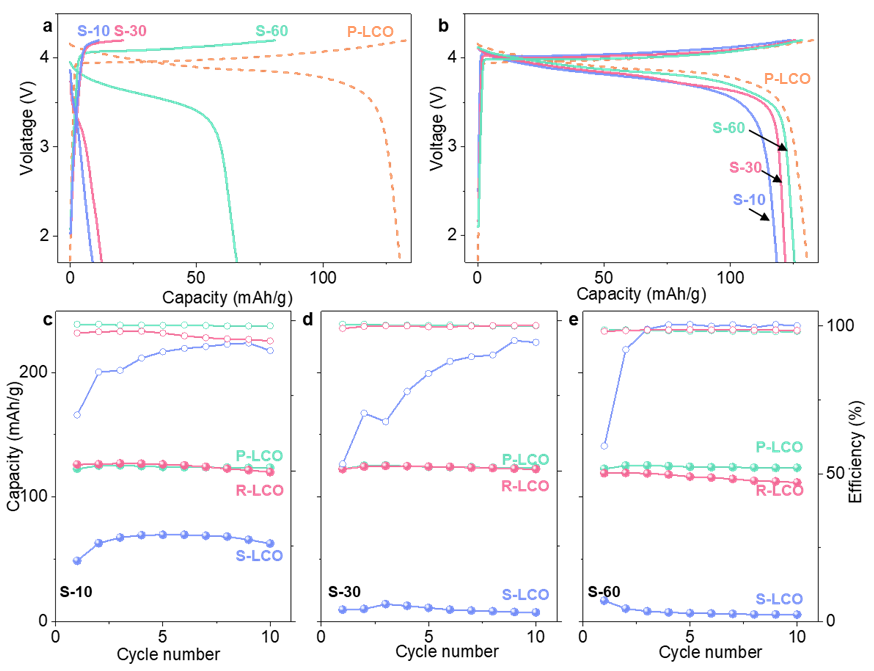


**Fig. 9 Electrochemical performance of different degraded LiCoO_2_ materials before and after repair. a**, Charging and discharging curves of degraded LiCoO_2_ before repairing. **b**, Charging and discharging curves of repaired LiCoO_2_ (S-10, 30, 60 where the number indicates the de-lithiation time, 10, 30, and 60 mins). Cycling performances of repaired LiCoO_2_ and the original degraded LiCoO_2_ with different de-lithiation times, **c**, 10 mins. **d**, 30 mins. **e**, 60 mins.


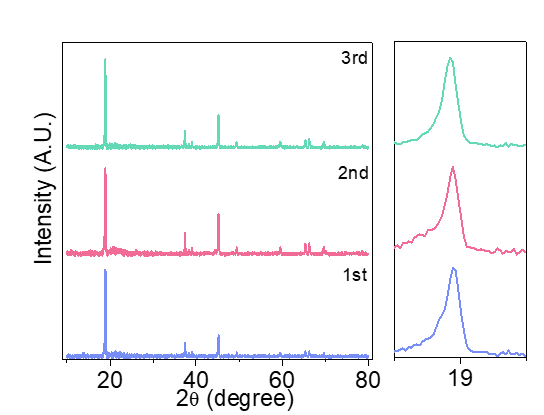


**Fig. 10** XRD patterns of repaired LiCoO_2_ using recycled DES for one, two or three repair cycles.


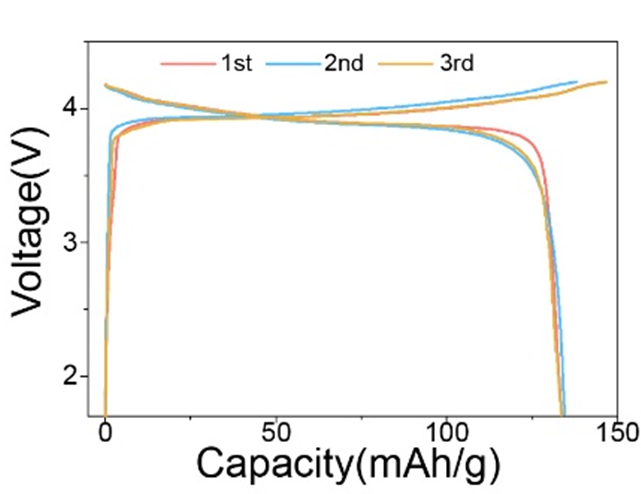


**Fig. 11** Charge and discharge curves of repaired LiCoO_2_ after using recycled DES for one, two or three repair cycles.


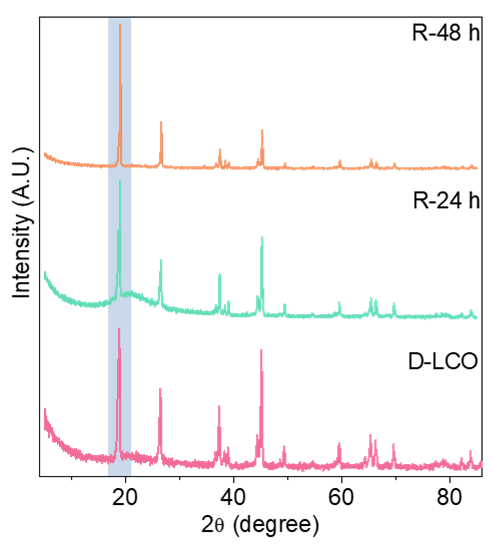


**Fig. 12** XRD patterns of degraded LiCoO_2_ treated in DES for different times.


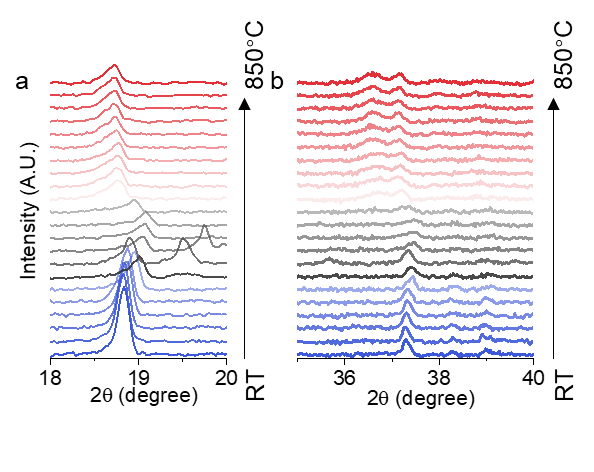


**Fig. 13** Specific *in-situ* XRD patterns of D-LCO-R at different temeperatures in different 2θ range: **a**, 18-20°, **b**, 35-40°. Only one characteristic peaks was observed before heated to 350 °C, and then a new characteristic peak related to (0 0 3) appeared and increased as temeprature rose. This phenomenon confirms the existence of phase transition.


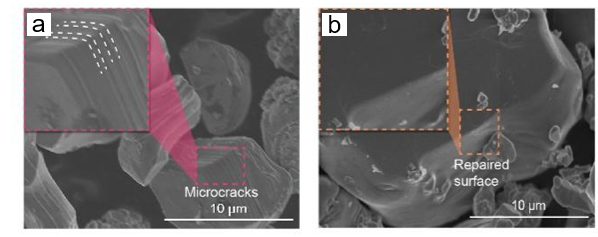


**Fig. 14** **a**, SEM image of D-LCO with microcracks at the edges. **b**, SEM image of D-LCO-R-H with complete surface.


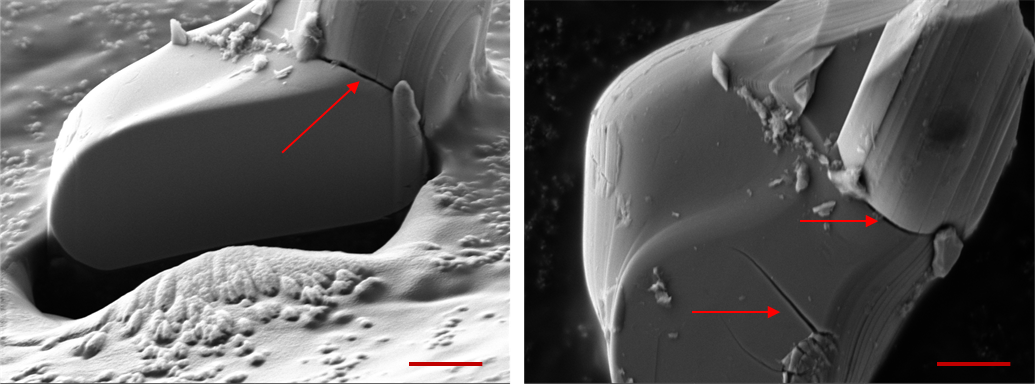


**Fig. 15** FIB-SEM images of D-LCO with microcracks at the surfaces.


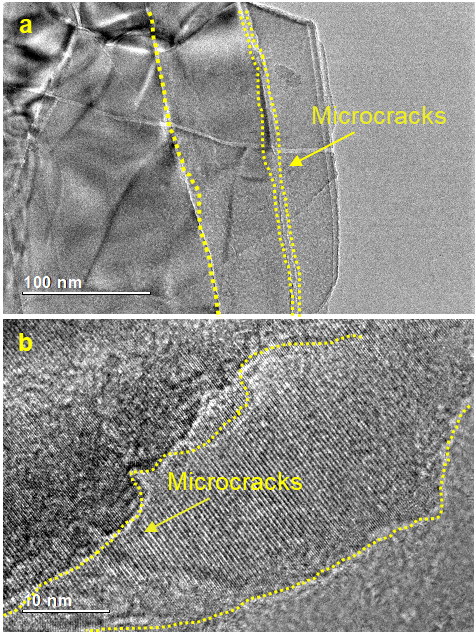


**Fig. 16** HRTEM images of degraded LiCoO_2_ with microcracks.


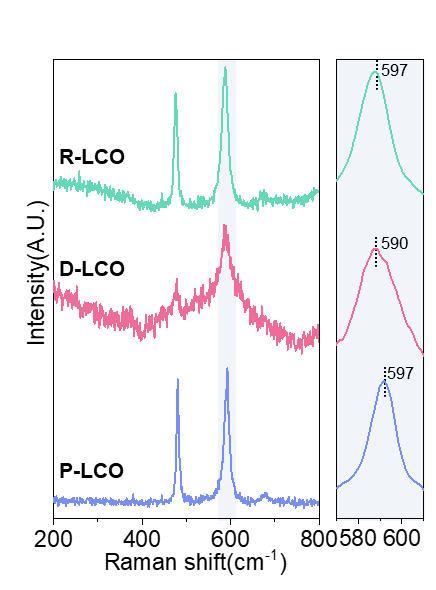


**Fig. 17** Raman spectra of degraded LiCoO_2_ before and after repair.


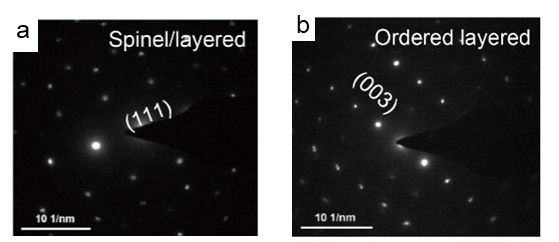


**Fig. 18** SAED patterns of **a**, D-LCO and **b**, D-LCO-R-H. Spinel type and ordered layered LiCoO_2_ are observed in D-LCO, while only ordered layered structure is seen in D-LCO-R-H.


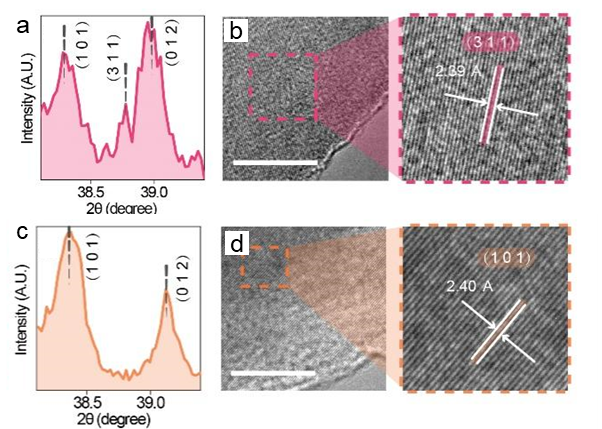


**Fig. 19 S**pecific XRD patterns of **a**, D-LCO, **c**, D-LCO-R-H, and the corresponding interplanar spacings, **b**, D-LCO, **d**, D-LCO-R-H.


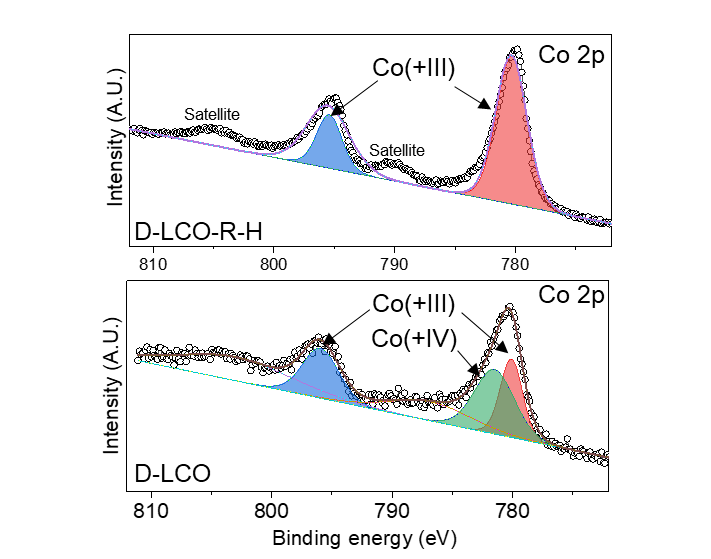


**Fig. 20** The XPS spectra of D-LCO and D-LCO-R-H. XPS spectra of D-LCO contains characteristic peaks related to Co^3+^ and Co^4+^, while only Co^3+^ was detected in D-LCO-R-H, indicating reduction of Co during Li^+^ intercalation.


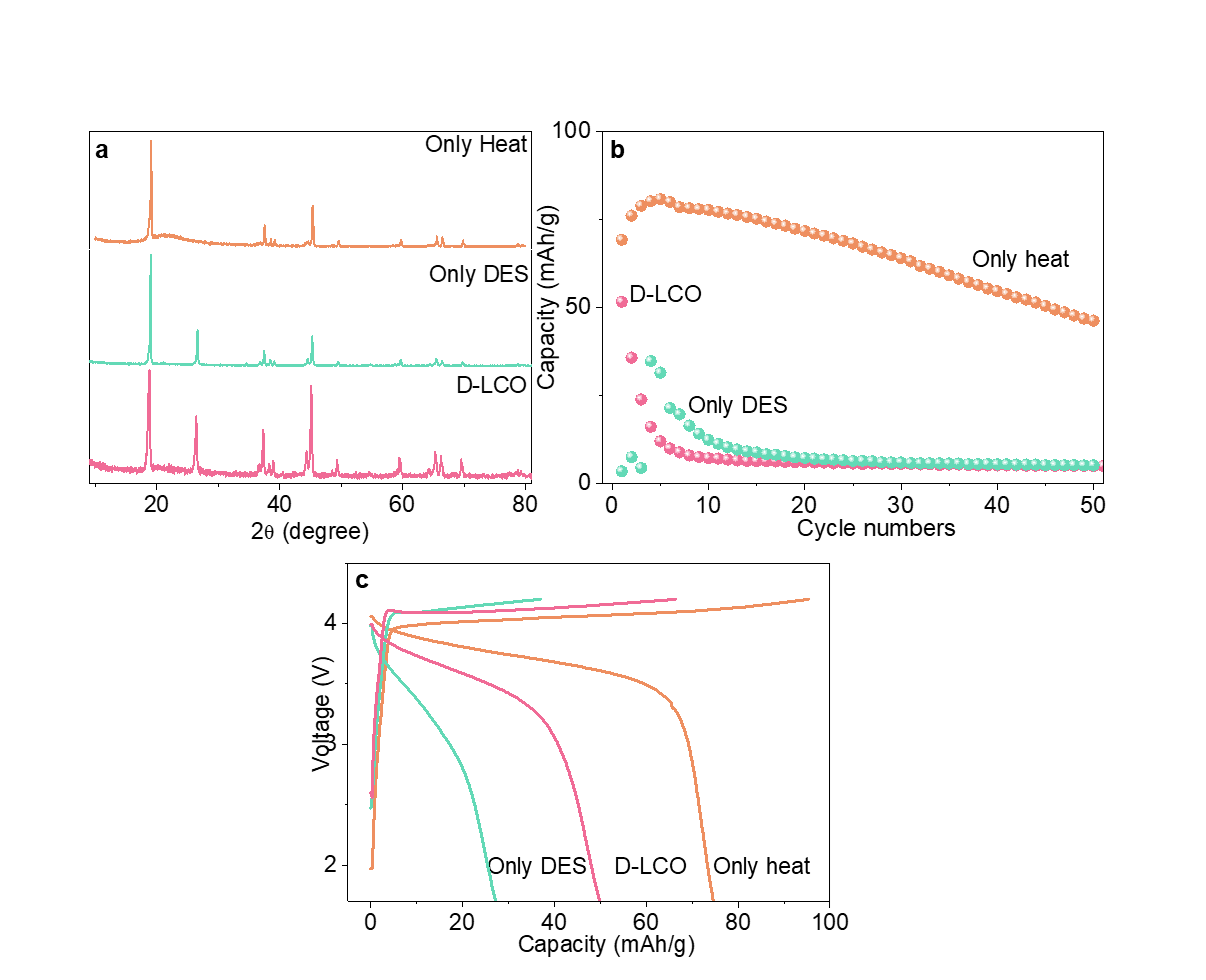


**Fig. 21 Structure and performance of repaired LiCoO_2_ using different treatments. a**, XRD patterns of degraded LiCoO_2_ and repaired LiCoO_2_. **b**, Cycling performance of degraded LiCoO_2_ and repaired LiCoO_2_. **c**, Charging and discharging curves of degraded LiCoO_2_ and repaired LiCoO_2_.
